# Early Decision: Effector and Effector Memory T Cell Differentiation in Chronic Infection

**DOI:** 10.2174/1573395509666131126231209

**Published:** 2013-08

**Authors:** Michael M. Opata, Robin Stephens

**Affiliations:** University of Texas Medical Branch, Department of Internal Medicine, Division of Infectious Disease, 300 University Avenue, Galveston, TX 77555-0435, USA

**Keywords:** CD4, CD8, human immune memory, infectious disease, malaria, mouse, T cells.

## Abstract

As effector memory T cells (Tem) are the predominant population elicited by chronic parasitic infections, increasing our knowledge of their function, survival and derivation, as phenotypically and functionally distinct from central memory and effector T cells will be critical to vaccine development for these diseases. In some infections, memory T cells maintain increased effector functions, however; this may require the presence of continued antigen, which can also lead to T cell exhaustion. Alternatively, in the absence of antigen, only the increase in the number of memory cells remains, without enhanced functionality as central memory. In order to understand the requirement for antigen and the potential for longevity or protection, the derivation of each type of memory must be understood. A thorough review of the data establishes the existence of both memory (Tmem) precursors and effector T cells (Teff) from the first hours of an immune response. This suggests a new paradigm of Tmem differentiation distinct from the proposition that Tmem only appear after the contraction of Teff. Several signals have been shown to be important in the generation of memory T cells, such as the integrated strength of “signals 1-3” of antigen presentation (antigen receptor, co-stimulation, cytokines) as perceived by each T cell clone. Given that these signals integrated at antigen presentation cells have been shown to determine the outcome of Teff and Tmem phenotypes and numbers, this decision must be made at a very early stage. It would appear that the overwhelming expansion of effector T cells and the inability to phenotypically distinguish memory T cells at early time points has masked this important decision point. This does not rule out an effect of repeated stimulation or chronic inflammatory milieu on populations generated in these early stages. Recent studies suggest that Tmem are derived from early Teff, and we suggest that this includes Tem as well as Tcm. Therefore, we propose a testable model for the pathway of differentiation from naïve to memory that suggests that Tem are not fully differentiated effector cells, but derived from central memory T cells as originally suggested by Sallusto et al. in 1999, but much debated since.

## INTRODUCTION

The phenomenon of memory is defined as the combination of the survival of antigen-specific T cells leading to an increased precursor frequency [1], and an improvement in the responsiveness of individual cells after an immune response. However, the relative contribution of each of these parameters is difficult to compare using monoclonal responses tested with transgenic T cells or MHC-tetramers, as in most studies, and so remains a matter of speculation. The intrinsic features sometimes seen in memory T cells include rapid production of cytokines upon restimulation [2-6], and the capacity to expand without engagement of costimulatory receptors, as on naïve B cells [7]. Memory T cells have also been shown to exist in a pre-activated state with more mitochondria [8, 9], allowing them to proliferate faster [10] with fewer cell cycle checkpoints [11], help B cells better, and reject skin grafts with the minor allogeneic determinant H-Y better than naïve T cells [12]. This improvement, however, may depend on continued stimulation; for example, it has been shown that long-term MHC contact is required for peak responsiveness and B cell help [12, 13], though it is unknown if this is due to persistent antigen, cross-reactive self or microbial peptides or low affinity peripheral contact with MHC [12-15]. Interestingly, a recent study also suggests that over time, CD8 Tmem lose functional avidity suggesting a mechanism other than deletion for the decay of protection, as also seen in MHC-deficient animals [16].

In order to achieve protection from a specific infection, the numbers of pathogen-specific T cells [17], and the levels of functional antibodies [18] must remain high; however, both seem to decline at least over long periods of time in the absence of antigen [1, 18-22]. Maintenance of all encountered specificities would require the repertoire to be able to expand to accommodate all encountered specificities at high levels and subsequent infections are reported to replace old specificities with new ones, leading to a decay for each infection [23]. However, expansion of the entire memory T cell niche may actually occur in some circumstances, such as seen in LCMV [24], though this may be due to a transient increase in the niche size in the context of the splenomegaly. Interestingly, in contrast to suggestions that larger precursor frequencies may lead to lower response rates, expansion of the memory pool by boosting actually improves responsiveness in subsequent challenges [25]. Given the shift towards an increasing proportion of memory T cells and the decay of the thymus with age, it is likely that an expanding memory niche plays some role in nature, however, it is not yet clear what factors determine if or how long memory cells for a given pathogen may survive, or be replaced by T cells of other specificities. Despite this slow decay, memory T cells clearly live longer than effector cells and are observed after vaccination and long past epidemics, proving that antigen-independent memory T cells and plasma cells do survive for long periods and protect us from various infectious diseases [1]. While both memory T cells and antibody producing plasma cells can survive in the absence of cognate antigen, including both CD4 and CD8 memory T cells, and even without interaction with MHC [26-30], studies have not been so definitive in the case of infectious antigens when maintenance of protection is the measured outcome [1, 18, 21, 31, 32]. It has been documented for several infections, including malaria [33], Leishmania, tuberculosis and helminth infections, that chronic infection or re-infection, actually reduces the pathological consequences of subsequent exposure [20, 34, 35]. This phenomenon has been termed premunition or concomitant immunity [36-41]. Illustratively, a short-lived *Leishmania* strain induced Tcm and some degree of protection in mice, but the best protection is induced by persistent parasites and Tem [31, 32, 38]. Similar findings in malaria and tuberculosis models show protective memory, and antigen-specific T cell responses decaying with time post-infection [21, 38, 42], though these decay times are much slower than those of Teff responses.

While there is data that people can remain protected from acute infections like measles and smallpox for many years in the absence of re-infection, in malaria, this protection is not completely penetrant in the population. Although 40% of people who had been exposed to malaria before its elimination in Madagascar 30 years before the study by Deloron *et al.*, were protected from re-infection, the other 60% were susceptible. This is in contrast to less than 20% protected among younger age groups [20, 43, 44]. This dynamic is more difficult to study for other chronic infections like tuberculosis and worms where exposure and occult infection is more difficult to quantify, and eradication has rarely been achieved even locally. Furthermore, even in the absence of new infections, absence of antigen is more difficult to prove *in vivo*. Studies that correlate this finding with a slow decay in the long-term survival of memory T cells and protection from vaccination have also been done (reviewed for malaria in [45] and [43, 46, 47]).

Zinkernagel argues persuasively that antigen-independent memory cells *per se* are not enough to provide protection from fast-dividing pathogens without the maintenance of highly responsive antigen-stimulated lymphocytes [18], suggesting that immunity, especially to chronic infection, is the combination of resting memory cells and activated effectors. The description of central and effector memory T cells by Sallusto and Lanzavecchia [48, 49] provides a framework for the division of labor suggested by this construct. Central memory T cells (Tcm) and effector memory T cells (Tem) are classified based on their phenotype and their functional and trafficking capabilities [48, 50, 51]. Tcm cells are defined by their surface expression of CD62L and CCR7, molecules that are coordinately regulated [52], and allow them to localize to the secondary lymphoid tissues and enter the T cell zone. CD4 Tem produce IFN-γ quickly, while Tcm make IL-2, and CD8 Tem are highly cytolytic [48, 53-56], but with low proliferative potential relative to Tcm [57-59], which have a greater lag-time to production of IFN-γ and are therefore measured in humans by a cultured ELIspot as opposed to an *ex vivo* ELIspot [60]. Recently, new subsets have been described that extend this paradigm to include a self-renewing memory precursor cell, and a long-lived tissue resident memory cell at each extreme of the spectrum. These subsets have been named stem cell memory T cells (Tscm), which appear less differentiated than Tcm [61]; and resident memory T cells (Trm), which remain in tissues with an activated phenotype post-infection [62].

Investigation of long-lived antigen-independent memory has largely centered on central memory, as the ideal candidate for a vaccine-inducible, long-lasting protection. This may be due to data suggesting that while Tem protect by virtue of their fast cytokine production (e.g. [63]), they have been shown to be short-lived [64, 65]; however, it has been difficult to distinguish Tem from short-lived Teff phenotypically, and therefore the literature is very unclear on the issue of how long Tem live [65], or how they are related to Teff [66-68]. Recent studies by the Harty group reported a population of CD27- effector/memory cells that undergo cell death over time, stabilizing the size of the long-lived memory pool [64]. The phenotype of these cells (CD62L^lo^CD27^-^) is similar to the late effector memory (TemL) subset first defined in human CD8 T cells [69, 70], although it may contain long-lived or continually generated Teff as well. While this mechanism is likely to contribute to homeostasis after infection, some conditions can alter their survival potential. For example, murine CD4 T cells with this phenotype can survive in conditions of chronic malaria infection [53]. The focus in the literature on resting memory T cells has led to attempts to reconcile the abilities of memory T cells to both survive in the long-term, and yet be readily activatable to perform effector functions. In support of this possibility, both Tcm and Tem survive after infection is cleared suggesting that both subsets can survive, and that while Tcm proliferate better, Tem maintain the ability to produce effector cytokines [48, 53, 54, 71-74]. However, recent data suggest that at the molecular level, this reconciliation between the tendency of activated cells to die and the need for their cytokines to protect, may not be possible for resting memory cells, as IL-12 and inflammation induces increased expression of T-bet, a factor essential for full Th1 effector differentiation, and at the same time, in CD8 T cells, reduces eomesodermin, a factor thought to regulate long-term survival potential [75]. A similar conclusion can be drawn from work on the cross-regulating pair of transcription factors Bcl-6 and Blimp-1, as well as Id2 and Id3; which control memory differentiation and effector fate respectively as opposing forces (Reviewed in [76]). Interestingly however, long-lived Tem express intermediate levels of Tbet, not the higher levels expressed by Teff at the peak suggesting there may be a balance allowing some level of both survival and protection [73, 74]. The concept of protection being mediated by a combination of cell types is supported by an interesting recent study that shows that both eomes^+^ (Tmem precursors and exhausted Teff) and T-bet^hi^ (Teff) T cells are required for protection [77]. Several other groups have also demonstrated that both Teff and Tmem contribute to control of chronic infection [71, 78-80]. The exclusivity proposed for the long-lived fate and Teff function is still being tested and is by no means certain (e.g. [81]); however if proven, it would mean that there is no way to generate long-lived memory T cells with heightened responsiveness. But if the Tem commonly being investigated are a mixed population including short-lived Teff, which confuses interpretation, then there remains the possibility that a population of Tem have different features that may allow them to maintain both an activated state and a longer lifespan than Teff. Therefore, it is critical to understand the relative contribution of short-lived but powerful Teff, and memory cells to protection in the case of chronic infection. Thus, defining the precursors of Tem in activation and differentiation is critical for informed design of vaccines for chronic infections including parasites.

## PROTECTIVE FEATURES OF TMEM ARE EFFECTOR MECHANISMS

Specific T cells to a given pathogen are numerically increased after a first challenge, and can be boosted by additional antigen exposure; and this increased precursor frequency reduces the lag time of the population in responding to re-infection [1, 82-86]. There is a lag time during infection for memory populations to be able to respond with proliferation to a second challenge [87]. This may be due to the refractory period for Teff to proliferate again and make IL-2 [88], combined with the slow increase in resting memory populations and slow decay of effector cell numbers [89]. There are several molecular mechanisms that enhance the responsiveness of memory T cells [7]. Memory cells are less dependent on accessory cell costimulation and can respond to many antigen-presenting cell types including resting B cells [48, 90-92], along with migration properties that make protection at peripheral sites better in a second exposure [51]; however, it is not clear which of these properties make Tmem protective, or if this may depend on the infection being studied [93]. Recent studies identified pathways that distinguish exhausted versus functional memory cells at the transcription level including differential expression in signaling pathways regulating quiescence and important transcription factors such as Eomes and T-bet [94]. While some correlates of T cell memory and protection have been discovered recently, we still do not have robust assays predicting protection for T cell vaccines under development. For example, it has been proposed that T cells producing multiple cytokines (IFN-γ^+^TNF^+^IL-2^+^) are predictive of protection in several infectious models [95]. Another interesting proposal that has been followed up recently is that the activation status of T cells is a good predictor of protection [22, 96]. This may be a proxy for which cytokines, and other effector molecules, they produce, as there seems to be a progressive acquisition of various cytokines along the spectrum of activation [97, 98]. The protective threshold for these cells is unknown, and more indicators, such as the presence of protective specificities, are likely to be required for robust readouts that functions as well as neutralizing titers for the antibody-dependent vaccines that we use. Furthermore, the correlation of extensive activation with protection [96] brings back the specter that protective T cell-mediated vaccines may require continual boosting, as Zinkernagel predicts [18]. Indeed, both memory and effector T cells have been shown to persist in humans [99], and Olson and colleagues have recently shown protection by a CD27^-^ effector population, which wanes over time in LCMV and *Listeria *[22]. Similarly, Sacks and colleagues observed that specific CD62L^lo^Ly6C+ Teff protect at the site of *Leishmania *infection (Peters NC, Pagan AJ, Lawyer PG, Hand TW, Roma EH, Stamper LW and Sacks DL personal communication). Interestingly, since the Teff in these studies do not have proliferative potential both to a primary and or in response to a second challenge, they also clearly distinguish the requirement of high secondary proliferative capacity from the ability to protect. Previously, it was shown that either cells that proliferate to high responding numbers (Tcm), or cells that have high functional avidity (Tem) can each protect in some systems and may be complementary in others [83, 84]. This correlates well with the type of decay of protection seen in mouse malaria and Leishmania as parasite decays [21, 31], and suggests that the increased protection seen during chronic infection may be due to effector cells and not Tem. Persistent antigen also increases both Teff and Tmem expansion and numbers [86]. This conclusion, if verified, would necessitate frequent boosting for full protection in infections requiring full T cell activity to maintain latency, however, it has not been ruled out that there are also longer-lived effector memory cells that are capable of maintaining effector responsiveness, especially given that Tem were defined at late time points as a subset by their markers and shown to have these “rememberance” characteristics [48, 54] and given the recent findings about long-lived resident memory cells in the tissues being antigen-independent [100].

The increase in antigen specificity in the memory pool may be the primary determinant of the speed of a secondary response, however, Tmem can also maintain enhanced effector functions making them even more protective. In order to understand what individual resting or long-lived memory T cells are intrinsically capable of, several groups have developed assays to test their “remembrance” characteristics, as Zinkernagel termed them [18]. CD8 memory T cells have been shown to proliferate faster than naïve cells and express higher levels of cyclin dependent kinase 6 and low p27^Kip1^ [11, 92], preparing them to pass the cyclin D3 checkpoint, but maintaining them in the quiescent state of G_0_/G_1_ cell cycle arrest. It was recently shown that CD27, *via* interaction with CD70 expressed by stimulated APCs, participates in maintaining this state *via* maintenance of phosphorylated Akt [101]. However, another study suggested that there is no decrease in the lag-time for division of memory T cells, but that they are immediately able to make IFN-γ on re-infection [102], which may be even more important for protection than expansion. Tmem have been reported to have increased signaling avidity compared to naïve cells, especially in the presence of inflammation [6, 103, 104], and some pre-activated memory cells, or long-lived effectors, maintain a partially pre-phosphorylated TCR suggesting a mechanism [14]. Similarly, Richer *et al*., recently reported that inflammatory cytokines influence the threshold of antigen required to trigger TCR and related molecule stimulation [103]. Therefore, even though both CD4 and CD8 Tmem can survive without contact with MHC [26, 28], they may not maintain high quality responsiveness in the absence of low-level stimulation, or “tickling” by MHC followed by homeostatic proliferation [12] to maintain this enhanced phosphorylation state. It will be difficult to determine which of these properties contributes to protection, and which memory phenotypes is contained in these properties, but it is essential that this type of work be carried out in clinically relevant systems to determine the characteristics that are vaccine inducible. Furthermore, while it is clear from the literature that survival of antigen-specific memory T cells *per se* does not require antigen, some mechanisms of intrinsic memory appear to be best maintained by continual stimulation, as are Tem [105]. However, it remains an open question how the subset known as effector memory T cells (Tem), which appear to be activated, are related to these actively maintained properties, as they have mostly been defined by their surface markers and location. Another important problem is how an activated subset could survive for the long term, given that the metabolism used by Teff, aerobic glycolysis, used for making enough new proteins, nucleic acids and fatty acids to divide quickly [9, 106], but leads to Lactic acid buildup and potentially an accumulation of oxidative damage; however, this is the metabolic state used by tumors to grow unhindered. While these studies suggest that antigen plays an important role in the maintenance of effector functions, we do not know the threshold of effector qualities that are necessary to protect for each infection. So, the possibility remains that there are long-lived effector memory T cells that contain sufficient protection to be advantageous as observed in CD8 T cells where malaria protective cells were detected after prolonged antigen presentation. In these studies, it was reported that protective memory cells develop after prolonged antigen exposure and expand better when transferred to initially immunized recipients as opposed to naïve hosts [86].

Recent studies have in fact identified a population with all of the traits presumed to be protective for localized infections. Following antigen clearance, a population of resident memory T cells (Trm) is maintained in the non-lymphoid tissues including lungs, gut [51] and skin [107]. Just like other memory cells (Tem and Tcm), they have low transcription of effector molecules [108], but can still provide enhanced protection compared to the T effector cells that circulate in the host [62, 107, 109] likely due to their localization at the site of infection from the earliest timepoints or re-infection [62, 110, 111]. Given that the best protective correlates to date are multiple cytokine production, localization to peripheral tissues and activation status [96]; features of highly activated T effector cells, it is important to understand how long-lived populations with these features could be generated.

## WHAT CAN EFFECTOR MEMORY T CELLS REALLY DO AND CAN WE GENERATE THEM BY VACCINATION?

As a population, Teff express the highest levels of effector molecules, suggesting that they are actually the most suited T cell subset for protection against infectious diseases, but they are short-lived. However, Tem have also been shown to express IFN-γ quickly, even into the memory phase, while Tcm express TNF and IL-2, and only regain IFN-γ after re-stimulation, as in a cultured ELIspot [48, 54]. CD8 T cells are generally predisposed to make IFN-γ, and this cytokine is regulated differently than in CD4, however, in order to measure long-lived CD8 Tcm, longer stimulation *ex vivo* are required for both CD4 and CD8 Tcm. Interestingly, Tcm were originally proposed to generate Tem based on the study of markers on CD4 human T cells *in vitro *[48], and in mice CD8 Tcm cells are shown to become protective Tem on infection with *Listeria *[112], however, current dominant paradigms assume that CD8 Tem become Tcm on withdrawal of antigen based on studies defining Tcm using only one marker to define Tmem, which is also expressed on Teff [79]. Other studies, however, suggest that these studies may be flawed due to the unphysiological number of TCR Tg T cells transferred and support a Tcm to Tem transition as seen in human T cells [113]. It is not clear how to rectify these seemingly contradictory assertions, though it could represent outgrowth of small, contaminating populations since Tcm are likely to be the most capable of self-renewal, and CD62L is notoriously shed *ex vivo* disguising Tcm in flow cytometry, or there may be a difference between CD4 and CD8 Tem. Tem have historically been defined as antigen-specific, CD44^hi^CD62L^lo^ or CD45RO^+^RA^-^CCR7^-^ T cells that can localize to peripheral tissues, however, Teff can also be CD44^int/hi^ or CD45RO^+^RA^-^. Therefore, most experiments are not able to distinguish Tem from acutely activated Teff and therefore Tem appear to depend on antigen for survival and function as Teff dominate the expansion phase population. While it has not been possible, in many studies, to identify effector memory as distinct from short-lived effector cells, the data suggests that most effector T cells are short-lived, and that once they down-regulate IL-7Rα (CD127), the majority die, both for CD8 cells where short-lived effector cells (SLEC) are defined by CD127-KLRG-1+ and for CD4. While Interleukin-7 and CD127 are important for memory T cell survival [114-116], the receptor is not essential, and memory cells can be formed in its absence [117-119]. Nevertheless, its expression has been cleverly used on day 8 of LCMV infection to identify memory-effector precursor (MPEC) CD8 T cells as distinct from short-lived effector cells (SLEC) in their ability to survive for the long-term as memory [120]. Unfortunately, this strategy does not work for CD4 T cells [74], but we have recently identified CD4 memory precursor effector (MPEC) T cells early in infection using the transient downregulation of CD127 on cells that have not yet changed other markers of activation to mark a subset of cells that differentiate into the memory lineage (M.M.O., Victor H. Carpio, Brian E. Dillon, R.S., unpublished data). It has recently become widely appreciated that there are CD127^+^ Tem, as well as Tcm [53, 120-122], but their longevity has not yet been established, other than in the tissues as Trm [62, 107, 123]. Until this distinction is made, it will not be possible to understand the contribution of either Teff or Tem independently to protection. Therefore, we hope that the use of the transient downregulation of the IL-7Rα on Teff to distinguish them from CD127^hi^ Tmem will help to clarify this important issue. There are two technical caveats to this method: one is that this down regulation is quite transient; and the other is, that Teff often appear to express negative amounts of CD127, however this can be remedied by increasing the voltage of the detector on the cytometer [53]. It remains to be seen if this strategy will work in the same way for CD8 T cells or in human T cells, but it seems likely, as they also transiently down-regulate CD127 though possibly with different kinetics than CD4 T cells [120, 124]. Further definition of memory T cell differentiation and effector cell activation pathways should help to clarify this puzzle and identify any potent cell that contains the elusive combination of long-life and intrinsic memory. Therefore, we will review the data for the pathway of generation of Tmem and explore the possible derivations of Tem.

## THE ORIGIN OF MEMORY T CELLS: MODELS OF DIFFERENTIATION

Several models have been proposed to explain the generation of memory T cells, but they generally propose either **a) **that events during antigen presentation determine early programming of cells to differentiate into memory T cells [125-128], or **b) **that events in the contraction phase such as differential susceptibility to withdrawal of growth factors and antigenic survival signals allow survival of a subset of effector cells into the memory phase [129-131]. These models are described as either early bifurcation of both Teff and Tmem from naïve T cells, or as survival of Teff that differentiate linearly over time into Tmem, allowing a later decision point. The contraction models generally consider Tem to be simply rested Teff, while early bifurcation suggests that Tem are less differentiated [132-137]. The contraction hypothesis suggests that some cells stochastically acquire durability for survival, despite the loss of stimulatory and growth factors in the contraction phase, for example due to higher expression of anti-apoptotic Bcl-2 [131, 138-140], and lower expression of pro-apoptotic Bim [141]. The increased death in this phase has been correlated to the reduction of antigen and IL-2 or increase in IL-7 or IL-7Rα in the phase of the immune response where the pathogen is cleared [131, 141-144], and this is the origin of the concept that Teff can “rest down” to become memory T cells. With the explosion of understanding of apoptotic pathways in the late 1990’s, it became apparent that while Bcl-2 was important for Tmem survival, and over expression prevents Teff death, neither exogenous Bcl-2 nor Bcl-xL allow generation of functional Tmem [140, 145], much as was subsequently found for IL-7Rα [117]. Subsequently, it has been persuasively argued that pro-apoptotic family members Bim and Noxa are responsible for the death of Teff [130, 139, 146, 147]. However, an interesting recent paper shows that while Bim deficient effector T cells do indeed survive the crash, they do not actually make quality functional memory T cells (similar to Bcl2 Tg “Tmem”). This suggests that the precursors of wild type, fully functional memory T cells are not found in the population that would normally die during the contraction phase [148], as also suggested by Prlic and Bevan [130]. Therefore, while contraction is surely controlled by apoptotic pathways including Bim, and although this phase does coincide with appearance of functional memory, contraction may not be required for the actual generation of memory cells (which requires TCR) though it may affect their survival (which doesn’t require TCR signaling, as described below). The contraction hypothesis has driven the field for many years, and has been reviewed extensively in other places [129, 141, 149, 150]. While it was always formally possible that early signals designate the population that become Tmem and that they express high levels of bcl-2 from the earliest timepoints, these early Tmem cells would not be easily detected within the much larger Teff population, as depicted in Fig. (**1**).

The contraction hypothesis provides a basis of interpretation for adoptive transfer experiments, which demonstrated that *in vitro*-derived effector CD4 T cells can progress into memory over time in the absence of further antigen stimulation [8, 26, 151, 152]. While 250 genes were found to be specifically upregulated in day 4 Teff compared to naïve T cells, 16% of these remained up in rested Teff on day 7, whether they were rested *in vitro* or *in vivo* in MHCII^-/-^ recipients, with memory associated genes gradually becoming expressed over longer time periods of rest [8]. However, it is not clear from these studies if all Teff can be Tmem, or if a smaller subset are contributing to the long-lived population. These studies also do not address the earliest timepoint at which effector cells can transition into memory, which might indicate if precursors were present all along, or only upon full activation of Teff. Interestingly, Opferman *et al. *did study this by activating H-Y-specific CD8 T cells, and suggest that memory precursors were detectable after five divisions [153]. These studies showed that memory T cells could be derived from cytokine producing effector cells, which has also been demonstrated in a more physiological system where LCMV derived *ifng*^+ ^or Granzyme B+ Teff generate both Tcm and Tem and long-lived Teff [154, 155]*. *Two new tools have been developed that will enable further exploration of the contraction phase and its relation to memory formation, transgenic mice with fluorescent markers for bcl-2 and a marker of caspase-3 activation [156, 157]. So far, these mice have not been used extensively to investigate early events, but it would be interesting to study, as it appears that only a subset of Teff is or remains bcl2^hi^ and has a strong memory precursor capacity, supporting an early derivation [156, 157]. Along these lines, experiments using a mouse that can be induced to express a permanent fluorescent protein on cells that are expressing CCR7 at the time of Tamoxifen treatment (r7UP mice), suggests that cells that express CCR7 at the peak of *Listeria* infection contain strong memory potential and are able to generate long-lived Tcm [73]. However further work using this unique tool will be important to understand this pathway. These studies have been taken to confirm that fully differentiated effector cells expressing these molecules are the precursors of memory T cells. However, only a subset of Teff are Tmem precursors able to differentiate into resting cells, and the timing of their appearance is difficult to test, though one study suggests that Tmem are preferentially derived from non-cytokine producing and presumably less differentiated cells [158].

## SINGLE CELLS CAN MAKE PHENOTYPIC HETEROGENEITY

**Fig. (1):**
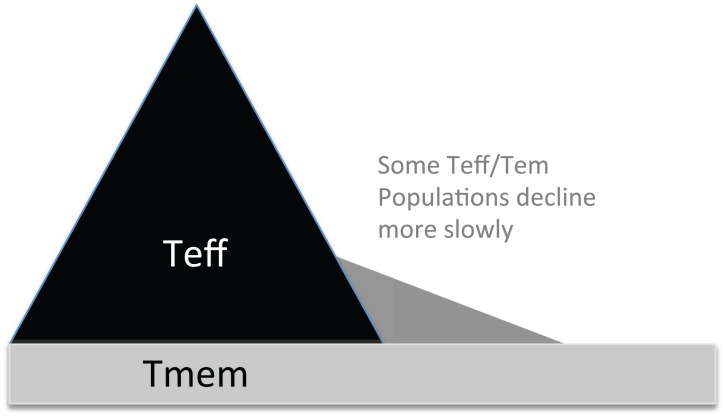
Effector T cells divide more and faster than Tmem, obscuring Tmem early in infection. The massive expansion of Teff has made early observation of Tmem challenging. Therefore, even with a stimulation that generates a fully asymmetrical T cell response, the Teff expand more eclipsing the original 1:1 ratio. Some Teff, such as CD8+ CD27-CD43-T cells, which include both CD127+ and CD127- populations, decay more slowly [22, 96, 120] creating the biphasic decay curve typically observed in T cell numbers post-infection.

The most significant recent observation to inform the question of when memory T cells appear in response to infection has come from developmental biology. In several developmental systems, dividing cells can be polarized to divide in an asymmetrical fashion into distinct daughter cell types [159]. Reiner and colleagues have painstakingly imaged both naïve and central memory T cells undergoing this process in some systems as much as 60% of the time [125, 160]. The two resulting daughter cells are phenotypically and functionally different as a result of the separation of effector proteins (IFN-γ, granzymeB as well as IFNγR, T-bet) towards the immunological synapse as directed by the signalosome and the MTOC, and their degradation by the proteasome in the smaller daughter cell at the other end [161]. Importantly, these elegant studies suggest that functional Teff and Tmem (or their precursors) can be derived in one moment, and that they are not selected by later events during Teff expansion or contraction, though inflammation can clearly influence the overall outcome [126]. Furthermore, the transfer of a single naïve T cell has been shown to generate any kind of heterogeneity that you look for including extent of division of individual clones and activation marker expression [162-168]. While these studies suggest that clones that generate smaller clonal families are likely to have a predominantly Tmem phenotype (CD27^+^ CD62L^hi^ KLRG1^-^) [164, 166], this could either be as a result of early programming by microenvironmental factors at the time of priming, potentially programming metabolic or cell cycle characteristics *via* Akt, p27^Kip1^ and mTOR [169, 170] or a result of active signaling. However, if it is programming, in this sophisticated study by Gerlach *et al*., it does not seem to be imprinted on an individual clone family for secondary proliferative capacity since tertiary stimulation seems to result in the maintenance of clone family sizes seen in the secondary response [164], importantly however, it is not clear that there is a change in numbers in a given family from a steady-state post-secondary timepoint to the tertiary response timepoint to test expansion. While Buchholz *et al. *supports the interpretation that individual clones generate all potential offspring; a major weakness in the study for our interpretation regarding a pathway of differentiation is the use of too few markers (CD27, CD62L without CD44 or CD127) to define pre-Tcm, pre-Tem and Teff. The scheme they use for their modeling therefore has not been validated, and we would argue that this precludes their conclusion that Tem are derived from Teff, including the linear pathway that they propose, which furthermore suggests that Tcm are gone by day 8 [166]. It would be interesting to use barcoding technology to follow individual cells as they expand *in vivo* to observe potential split phenotypes during a response as predicted by asymmetric division. Similar experiments done *ex vivo* by Lemaitre *et al. *resulted in the observation of some clones that do indeed generate two types of offspring in single cell culture [167], though the study also was not designed to observe complete Teff and Tmem phenotypes. This body of work suggests that diversity may not only come from the actual first division, since that would only allow two fates, but that at each subsequent division [167], additional diversity is added. Further study needs to be done to establish the order of events and pathways of activation and differentiation for naïve cells. The results of Gerlach and Buchholz suggest that each individual naïve T cell responds to stimulation differently *in vivo*, likely depending on the variables of the particular antigen presentation events that it experiences in the first 48 hours. They suggest that because each family tends towards expression of activation markers differently, with all families collectively leaning towards Teff markers on day 11 as expected, that Tmem derive from Teff. However, since a large family that is mostly KLRG1^+^ or CD62L^lo^, still has some KLRG1^-^ or CD62L^hi^, non-terminally differentiated cells, there exists the possibility that on day 11, when the snapshot is taken, the Teff dominate, but Tmem (or MPECs) exist within each clonal family. The rough correlation with larger families and expression of T-bet and eomes supports this as well [166]. For example, stronger stimulation in a particular cell’s microenvironment may generate a large Teff population that potentially obscures Tmem within that same clonal family, as in the population at large (as in Fig. **1**). Their own previous data suggests that indeed all clones do actually produce both Teff and Tmem, consistent with asymmetric division [163]. Furthermore, although pathogen dose was titrated, even the lowest dose of bacteria is likely to generate T cell competition and a limiting number of APCs with peptide in an ideal state of activation. It would be interesting to test this hypothesis using an optimal combination of antigen dose and inflammatory stimulus, and small but traceable precursor numbers to attempt to prevent significant variation in presentation, perhaps using identical “bar coded” or surface-marked clones to follow the response through time. By developing this system, one may be able to generate several identically sized families at a given dose. Fig. (**1**) also highlights the predominance of Teff than Tmem at any given effector timepoint, which some have contended annuls the possibility that one naïve cell can make one Teff and one Tmem. Since Teff proliferate much more than Tmem, and Tmem precursors can be recruited into the Teff response until antigen is eliminated, Tmem could exist at much lower levels than Teff from the earliest times of an infection, only becoming apparent after the contraction, even if they had been pre-determined earlier.

**Fig. (2):**
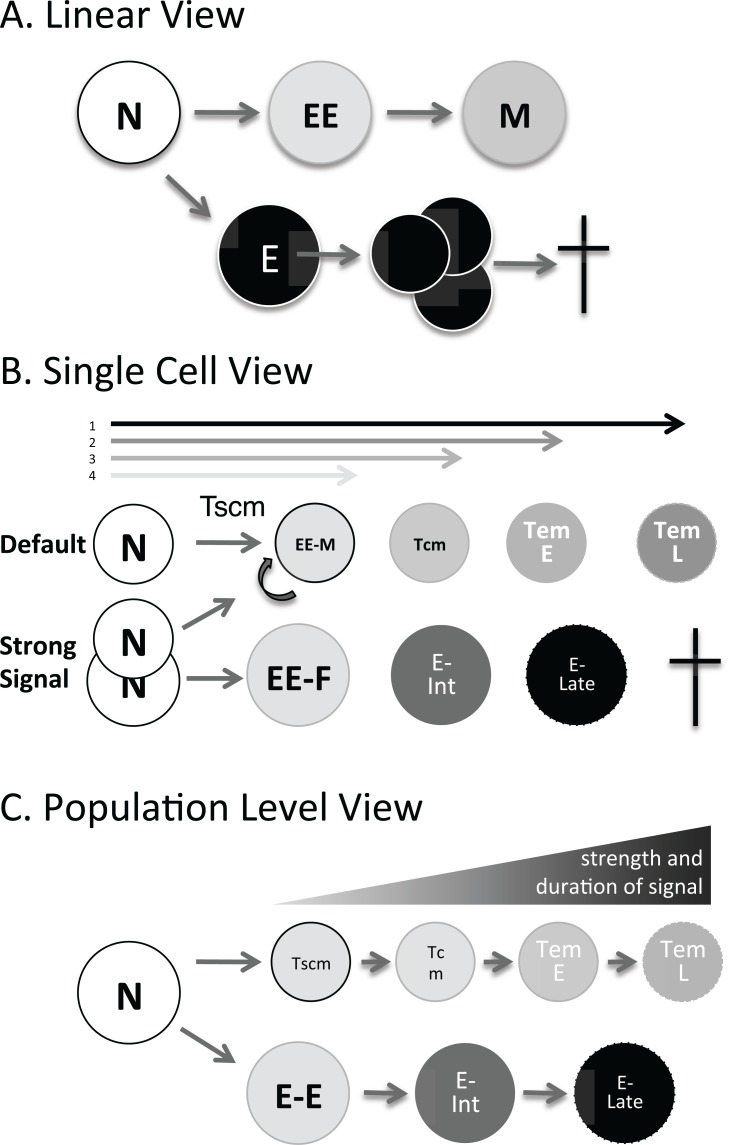
Evolution of a model of Effector memory differentiation three views. A. The linear view suggests that Naïve T cells make early Effectors which make Memory, while mature effectors expand quickly, and die. CD8+ EE (and MPEC) and E (SLEC) are identifiable at the peak of infection [120, 175]. B. Each naïve T cell integrates it’s own signals (T cell receptor, co-stimulation and cytokines) over the lifespan of the antigen presenting cells with specific antigen. A strong integrated signal determines if a naïve T cell divides asymmetrically and generates both a Tmem and a Teff precursor or undergoes default programming to become memory. It also determines how many times a particular clone divides and what the phenotypic outcome is for that clone with asymmetric division potentially ensuring heterogeneity at each division. C. At the population level, considering all clones, populations purified by phenotype behave as shown with a linear pathway of differentiation from Tcm > Early Tem > Late Tem, and Early Effectors to later Effectors that die. The predominance of a given population at a given timepoint is determined by the collective strength of signal over time.

It has recently been demonstrated by King *et al. *that the strength of T cell signaling determines whether a cell undergoes asymmetrical division or not [165]. Therefore, a strong signal may lead to asymmetric division and one fast dividing Teff and one slow dividing Tmem; while a weak signal may only lead to two Tmem, or early effectors which can become Tmem. This elegant and unique study suggests a striking new hypothesis for the effect of low doses of antigen on Tmem differentiation which explains previous data in a new way [163]. Memory differentiation in CD8 T cells can actually be induced with a mild signal that induces little Teff generation and has been called the default pathway [87, 126, 171]. In order to reconcile several points of view, we present several complementary models of Teff and Tmem differentiation in Fig. (**2**). These three illustrations actually represent the same bifurcating pathway where naïve T cells divide to generate both Teff and Tmem precursors. Therefore, we propose that while memory differentiation often appears linear as shown in Fig. (**2A**), with Teff capable of generating Tmem, and Tmem appearing after contraction, this can be the result of a subset with Tmem precursor potential (MPEC) differentiating early upon infection, while other Teff (SLEC) proceed to expand exuberantly, and then die. If one investigates the problem from the point of view of a single cell, the varied strength of the integrated early signals in each cell could generate some less activated pre-memory cells as well as cells that were destined to divide a lot and die, Teff, assuming that cells that turn over more slowly, generate fewer offspring, and are less close to terminal differentiation are Tmem (Fig. **2B**). This pattern of clonal expansion (beyond 50 clones [164]), adds up to the sum that we see from the perspective of the heterogeneous phenotypes in the whole responding population (see Fig. **2B**,**C**). We will come back to these models again later.

## DECISION POINT FOR EFFECTOR AND MEMORY DIFFERENTIATION IS DURING ANTIGEN PRESENTATION

Although Tmem only dominate the response after the contraction of Teff, evidence is steadily mounting that programming of memory cells is actually predetermined during the early stages of antigen exposure by the combined strength of incoming signals to the T cell [126, 172-177]. This signaling is integrated during the extended contact up to 48 hrs. of naïve T cells with antigen presenting cells [87]. Some immune responses generate a large proportion of proliferating effector T cells which undergo cell death in the contraction phase [127, 178, 179], while other responses quickly generate a readily apparent cohort of memory phenotype T cells which survive longer [128, 172, 180, 181]. Additionally, other studies have shown that different types of stimuli can make a T cell response entirely dominated by the generation of memory T cells [126, 128, 182]. This has been described as an indication that the memory phenotype is a default pathway on T cell activation [126], and demonstrates that differentiation of Tmem does not depend on the contraction phase [172]. Furthermore, many infections have been shown to generate a detectable cohort of Memory Precursor Effector T cells (MPEC), first identified by Kaech *et al.*, at the peak of CD8 T cell expansion even before the contraction phase [120]. This alone supports the conclusion that MPEC are either selected or “programmed” before the contraction of the T cell response. The authors conclude however that MPECs undergo an effector to memorytransition due to increased IL-7 and IL-15 that promote the survival of transferred MPECs. However, the predominant phenotype CD44^hi^CD62L^hi^CD27^+^CD122^hi^Bcl2^hi^ and 30% KLRG1^-^ of the transferred CD127^+ ^cells suggests that they may already contain Bcl2^hi ^memory T cells [120]. Interestingly,previous studies by the same group actually suggested that the initial stimulus programmed the differentiation of Tmem [173]. Furthermore, in the influenza model, KLRG1^+^CD127^-^ CD8 T cells can revert and survive as well [183]. Early appearance of CD4 pre-memory T cells has also been demonstrated as early as day 3 post-infection in *Listeria *[73] and in influenza infection by day 3.5 [184]; and phenotypic Tmem have been shown to be generated even in the presence of chronic viral and parasite infection [53, 185]. The elegant study by Pepper *et al.* demonstrates that these early CD4^+^CCR7^+^CXCR5^+^ Pre-Tcm at the peak of infection go on to generate central memory T cells, while Marshall *et al*. show that memory precursors are contained within PSGL1^+^Ly6C^hi^ CD8 T cells [73, 74]. In our view, these studies present an important contradiction of contraction theory, which presents the challenge to understand how Tmem, especially Tem with effector functions, can be made without an activated Teff stage. As reviewed below, the identity of factors that determine the differentiation fate of activated T cells towards Teff or Tmem has recently attracted a considerable amount of attention and several authors have performed various studies demonstrating that the events following early stages of antigen encounter play an important role in regulating the memory and effector fate of responding T cells.

## STRENGTH OF TCR SIGNAL IN MEMORY T CELL DIFFERENTIATION

It is apparent from the literature that the combined strength of the three T cell activating signals, namely TCR signaling, costimulation (signal 2), and inflammatory cytokines (signal 3), play an important role in the fate decision of responding T cells [186, 187]. Experiments using affinity altered peptide ligands (APL) or mutated MHC to investigate effects on proliferation of memory T cells [173, 188-190], indicate that a weaker TCR signal favors memory generation over Teff expansion. Using a system that allows full or abortive antigen exposure in a bacterial infection, Prlic *et al. *reported that stimulation of CD8 T cells for a shorter time promoted the development of functional memory over expansion of Teff [127, 188]. Similar findings have also been observed in other systems [127, 173, 190]. Experiments to determine the role of antigen avidity in differentiation have shown that while stimulation with low avidity peptides can activate T cells to proliferate, low affinity responders expand as well as T cells with high avidity [189]. Furthermore, the expansion of low avidity T cells is shorter, changing the contraction kinetics. The result is that cells exposed to stronger signals are apparent for longer and develop into functional memory explaining how the repertoire increases avidity over time into the memory phase [189]. In contrast, Teixero *et al. *mutated the TCRβ transmembrane domain disrupting the immunological synapse and NFκB activation and observed that this mutant signal generates phenotypic Teff, but does not induce expansion or produce long-lived memory CD8 T cells, while the wildtype TCR generated both. In a study where TCR expression was reduced by up to 30 times, Teff and Tmem phenotypes were not affected [191], and TCR itself is dispensable for Tmem survival [192]. Other studies corroborate this finding using mice deficient in MHC or signaling molecules [26, 27, 105, 115]. However, in these studies, lower cell numbers were generated [193]. These elegant studies nevertheless suggest that strong synapse formation and asymmetric division are important for the generation of memory, as recently reviewed [194], and developed below. Similar studies in the CD4 system show that low frequency CD4 T cells have high chances of making memory T cells that survive as compared to higher frequency CD4 T cells [195]. This suggests that reduced naïve precursor numbers allows for strong or long-lasting contacts with antigen presenting cells, improving the development of memory for a few cells, or increasing the subsequent numbers of asymmetric divisions for a given clone (see King *et al. *below).

While there is not as much information about the effect of TCR avidity and overall antigen presentation strength on CD4 T cell expansion and memory differentiation, several studies suggest that avidity does play a role. Some interesting work has been done observing the expansion of low and high affinity T cell clones using model peptides [196, 197]. Using either the high (5C.C7) or low (2B4) affinity TCR Tg mice, or by sequencing junctional diversity to identify the fraction of high (Jβ1.2) or low (Jβ2.5) affinity TCRJβ segments in the population of clones in the response at the peak and later time points, it appears that high affinity clones disproportionately enter the memory pool (and respond in the secondary stimulation) [198]. Interestingly, Baumgartner *et al. *suggest that the affinity of the TCR may affect the slope of the contraction of low affinity T cells as suggested above for CD8 T cells [189, 197].

## ROLE OF STRENGTH OF CO-STIMULATION IN MEMORY T CELL DIFFERENTIATION

Other molecules in the immunological synapse have also been shown to regulate activated T cell fate. For example, the integrin CD11a which stabilizes the immunological synapse with CD18 by interacting with ICAM-1 during T cell activation [199], regulates differentiation as well as promoting T cell proliferation and cytokine production [200]. T cells deficient in CD11a tend to differentiate towards Early Effector cells and MPECs as opposed to Teff [201], suggesting that CD11a engagement, and perhaps the durability of the immunological synapse contributes to the strength of signal regulating the differentiation choice [202]. In support of this interpretation, generation of memory is diminished in ICAM-1 deficient mice as well [203], and *Icam1*^-/-^ T cells do not polarize or divide asymmetrically [125]. Mittrücker *et al. *reported that while expansion of Teff is severely reduced in response to *Listeria*, in CD28^-/-^ mice, a significant cohort of longer surviving memory T cells is generated [204]. Compton and Farrell observe a similar phenomenon in *Leishmania* infection, however they show that the animals are not protected from a second infection suggesting that these Tmem may not be functional [205]. This observation may also be due to differential cytokine production in CD28^-/-^ mice in response to *Leishmania*, which requires a strong Th1 response as Th1 differentiation is also affected by differential co-stimulation and strength of signal [206]. CD28^-/-^ mice did generate Tmem in response to LCMV and were protected, though numbers of responding T cells were lower [207]. Other molecules required in the immunological synapse for complete T cell activation such as the signaling molecule downstream of the TCR and CD45, Lck, also affect T cell fate. It has been shown that Lck^-/-^ T cells produce less IL-2 and have impaired cytotoxic effector function. Furthermore, T cells from Lck^-/-^ mice primed with influenza upregulate CD44 normally and survive longer, characteristics associated with memory T cells [208].

Cytokines, the third signal in T cell activation, have also been shown to play a role during early stages of activation on cell memory differentiation. IL-12, produced by APCs during antigen presentation in response to innate inflammatory signals preferentially induces the generation of effector but not memory CD8 T cells in various infections [149, 209-212]. Pearce and Shen showed that IL-12 deficient mice infected with *Listeria* generated robust CD8 memory T cells that greatly protected against re-infection. More memory precursors are formed in the absence of IL-12, which expand as they self-renew. Strikingly, upon re-stimulation with IL-12 *in vitro* for 3 days, naive cells produce more IFN-γ and reactive oxygen species (ROS), characteristics associated with effector function [211]. Furthermore, it has been shown that direct IL-12 signaling in CD8 T cells determined the generation of short-lived effector cells or long-lived memory precursors [210]. Recently, Rao and colleagues provided a mechanistic synthesis when they reported that when mammalian target of rapamycin (mTOR), which regulates cellular metabolism and is maintained by IL-12, is blocked, *tbx21* expression (which promotes generation of Teff) is decreased while Eomesodermin (Eomes) is increased, promoting generation of memory precursors [213, 214]. Interestingly, the role of IL-12 and T-bet in Teff fate may be primarily at the level of controlling the numbers of Teff that expand [74]. Other transcription factors also play a role in the effector/memory fate decision including the ratio of B lymphocyte-induced maturation protein 1 (Blimp-1) and B cell lymphoma 6 (Bcl-6) [176, 215, 216]. Interestingly, Blimp-1 promotes generation of functional effector cells, as it does in B cell activation promoting plasma cell generation [217]. Even though an intact population of phenotypic memory develops in the absence of Blimp-1, it also plays a critical role in their conversion to protective cells during a recall response [215]. Blimp-1 antagonistically affects fate decisions regulated by transcriptional repressor Bcl-6, with higher Blimp-1 expression inducing terminally differentiated effector T cells while high Bcl-6 promotes Tcm and MPECs with high proliferative potential in both CD4 and CD8 T cells [216, 218, 219]. This has been shown to be due to the ability of Blimp-1 to activate important developmental genes that Bcl-6 represses [132], however, it is not clear how early post-infection the ratio of Bcl-6 and Blimp-1 is determined, or how much it is affected by cytokines, though IL-2 has been reported to play a role in Bcl-6 transcription *via* STAT5 [220]. Studies by Manjunath and colleagues *in vitro *showed that the amount of IL-2 present during the initial stimulation generated two different types of responding CD8 cells, with high dose IL-2 inducing immediate cytotoxic effector cells, while low doses of IL-2 generated T cells with low levels of cytotoxicity that were capable of surviving longer. The long-lived cells were also observed at all tested concentrations of IL-15 [181]. While IL-2 promotes the expansion of Teff [73, 221], IL-7 and IL-15 promote memory. CD8 memory precursors that do not require high doses of IL-2 for their generation [175], but they do require CD4 T cell IL-2 for their persistence [222]. IL-2 and its receptor CD25 play an important role in establishing reactive memory CD4 T cells in an IL-7-dependent manner, which suggests that IL-2 and IL-7 signals are separated in time and perhaps space as well [223].

Interestingly, CD8+ T cell activation involves a metabolic change to increased levels of glycolysis and a switch from this state to fatty acid oxidation has been shown to be essential for the development of Tmem [9]. Generation of Tmem or their survival through the contraction phase seems to be controlled by the central metabolic sensory pathways emanating from mTOR and AMPK [224]. Withdrawal of IL-2 after acute T cell stimulation coincides with T cell contraction, and also with the switch of T cell metabolism from the anabolic aerobic glycolysis to the catabolic fatty acid oxidation of memory cells. In the absence of TRAF6, which signals through AMPK, withdrawal of IL-2 does not lead to a switch to fatty acid oxidative metabolism and very few memory T cells persist in a T cell specific mutant of TRAF6 [169], suggesting regulation of this metabolic switch by external signals. This switch can be promoted with metabolic drugs that target mTOR and induce fatty acid oxidation providing hope of a new class of “adjuvants” [169]. These studies also suggest that the formation of memory phenotype cells is regulated past the initial stimulation by growth factor or cytokine signals and potentially by environmental factors, such as nutritional status or side effects of infection (e.g. anemia, acidosis and hypoglycemia in malaria) which could modify survival of memory cells.

## MAINTENANCE OF MEMORY BY CYTOKINES AND INFLAMMATION

Acute cytokines (like TNF and IL-1) affect the strength of the response *via* recruitment of antigen presenting (and other innate) cells into the response, and induction of appropriate costimulatory molecules on them. Cytokines produced by APCs like IL-12 and type-I IFNs can regulate T cell differentiation, while other cytokines are involved in later stages of memory T cell survival. IL-7, IL-15, and IL-21, made by stromal cells, are implicated in survival and differentiation of memory T cells with IL-7 promoting homeostatic proliferation and survival as opposed to being the sole determinant of differentiation down the memory pathway [142], and IL-15 (induced by IFNα) promoting Tem or Teff expansion [225-229]. Interestingly, a recent report suggests that IL-7 and IL-15 *in vitro* can lead to the differentiation of stimulated naïve T cells into Tscm, suggesting that this early memory precursor is directly derived from naïve T cells [230], and although intermediates were not studied in this work, that is the model proposed by workers in the Tscm field [61]. Interestingly, STAT3 has been shown to be important for survival of CD8 memory T cells as well, likely indicating a role for IL-21 and/or IL-10 in the maintenance phase of the memory “lifecycle” [231, 232]. IL-7Rα expression of CD8s during infection has been shown to be regulated by Gfi-1 and GABPα, however it is not yet clear if these factors also regulate functional fates [233]. The transcription factor Id3 is specifically upregulated in MPECs [234], but is downregulated by Blimp [235]; while Id2 is involved in both development of Teff and their survival into the memory phase with Id3^hi^ marking pre-Tmem very early in infection [234, 236-238]. Involvement of the Id family transcription factors is interesting given that they are downstream of important developmental signals like the BMP family and the IGFR family, but the connection to cytokines or early events has not yet been made, although TNF and NFκB can play a role in Id2, and Id3 regulation in neuroinflammation [239]. Other studies have reported involvement of Foxo1 in the regulation of differentiation and survival of Tmem [240, 241], and is inhibited by IL-2, suggesting that this may be an early event. Interestingly, mTOR and Akt, which integrate signals from growth factor and cytokine receptors to mediate survival by regulation of transcription and metabolic enzymes, are also at the center of the network of factors determining the fate of activated T cells [213, 242]. As discussed above, inflammatory cytokines like IL-12 and Type I IFNs also play a role in the decision point during the presentation of antigen, so likely at the initial point of contact, not in the long-term survival phase. In human CD8 T cells, IL-12 induces Tem-like differentiation while IFN-I promotes Tcm formation [243], this could suggest that distinct antigen presenting cells, or priming environments favor one over the other subset. Continued production or stimulation of cytokines and chemokines also plays a role in the maintenance of Teff and Tem recruited to peripheral tissues [244], as does antigen [86]. Some interesting studies also suggest that the functional capabilities of these protective cells are enhanced by the cytokines IL-15 and Type I IFNs [245, 246], which could be generated by un-related stimuli to maintain Tmem functionality. These studies and others suggest that the maintenance of memory T cell numbers and functions beyond the contraction phase depends on cytokines in their environment conducive to their survival as well as cytokine stimulation and MHC to maintain their homeostatic proliferation and enhanced “remembrance” functionality, and reduce autoimmune memory specificities. As many previous studies of Tem may include short-lived Teff in the analysis, it will be interesting and important to determine what immediate effector functions a more pure Tem (CD127^hi^CD62L/CCR7^lo^) population is capable of and what role antigen plays in maintaining their numbers and intrinsic capabilities as they relate to protection.

## THE ELUSIVE SOURCE OF EFFECTOR MEMORY T CELLS

Various groups have proposed development of Th1 memory from Th1 effector cells as in a progressive differentiation model, as described above and in an extensive literature on Th1 differentiation and commitment (reviewed in [247]). While there seems to be an intrinsic contradiction between being long-lived and maintaining effector functions, we have been exploring Tem as the potential cell type capable of balancing these two features. It has been proposed for both CD4 [177, 73] and CD8 [175] that early effector cells are enriched for memory precursors in model antigen [177, 73] and viral infection [74]. We have uncovered a model of differentiation of specific CD4 effector memory T cells using the *Plasmodium chabaudi* mouse model of malaria suggesting that this is also the precursor for Tem. We have strictly identified this Teff^Early^ subset phenotypically and found that while it contains cytokine-producing cells, as suggested by previous studies showing Tmem differentiation from cells capable of effector function [8, 152, 154], this population also has a high level of Bcl-2 expression [73]. Interestingly, these multi-potential cells can make Tem, not *via* an effector activation pathway, but *via* Tcm, potentially even in the absence of a further activation event (M.M.O., Joshua M. Obiero, Brian E. Dillon, Jordan C. Carl, R.S. unpublished observation). This suggests that Tem may indeed be long-lived, like Tcm, as originally proposed by Sallusto *et al.* [48], although it is challenging to distinguished Tem phenotypically from Teff. If this is the case, then Tem may proceed down a slow differentiation pathway, as opposed to being the products of extensive activation and division in the expansion phase, explaining the observation of shorter telomeres in Tem compared to Teff [48]. Our model, supported by the original data from Sallusto for human CD4 Tcm and by Huster and Busch for murine CD8s [112], is shown in Fig. (**2**, all three panels show the pathway from different perspectives) and proposes that Tem are predominantly derived from early Teff and Tcm, not from progressively differentiating Teff. Many of the studies cited in the previous section, suggests that even during the expansion phase, specialized immature memory precursors exist. These precursors are likely to be included in the MPEC population observed at the peak of infection [73, 125], and may resist the dramatic expansion seen in the rest of the Teff population, as suggested in Fig. (**1**). This could also be envisioned as a migration phenotype whereby long-lived memory precursors potentially seek sites of low antigen stimulation, or resist re-activation in other ways, such as inhibition of antigen-receptor signaling. Supporting this concept, Serre *et al. *have shown that early Tcm recirculate [248].

The combined force of the data reviewed here, as well as our own observations in *P. chabaudi*, have led us to propose a new model of memory T cell differentiation (Fig. **2**). This model can be seen from several perspectives. A linear pathway of differentiation necessitates the effector cell precursor arising early in the immune response as the precursor of memory, since later Teff proliferate very fast and die (Fig. **2A**). While it is possible that more mature Teff contain memory potential, the progressive differentiation model [70, 97, 249, 250] would predict that they would contain less memory and proliferation potential than earlier or less stimulated [177] cells. This model of differentiation and the phenotypic markers used to distinguish the subsets on the continuum are nicely reviewed for human and non-human primate T cells in Mahnke *et al. *[253], but a similar path for effector cells is largely undescribed for mice. From the perspective of an individual cell, such as those studies using single cell transfer [166], or single cell marking by DNA sequences or surface markers [164], it is apparent that each naïve T cell, even if they have the same TCR specificity, are subject to variations in microenvironment and signals 1-3 which appear to affect their fate, with each clone and its proliferative “family” combining to make a heterogeneous population. We propose that the combined strength of signal that each naïve clone is exposed to in the antigen presentation milieu can lead to more or less differentiation, including more or less asymmetric divisions, leading to differing ratios of Tmem and Teff among the progeny of each clone. Stronger signals, appear to promote Teff generation (and large clonal families at the peak of expansion, and more asymmetric division [165], while less strong signals may promote smaller expansions of Tmem, as shown in Fig. (**2B**). The combined effect of this process at the population level is shown in Fig. (**2C**), where Teff and Tmem fates are determined during contact with antigen presenting cells making more or less Tmem, which divide more slowly but remain for longer periods for increased antigen-specificity and surveillance against further insult. Although several aspects of the model appear to hold for both CD4 and CD8 T cells, for example asymmetric division, and Early Teff as MPEC or pre-Tcm, as well as signals that can generate Tmem without Teff, it is not yet clear if the model applies to both.

## THE QUEST FOR THE FOUNTAIN OF YOUTH

The possibility of finding a vaccine-inducible memory T cell population that contains the ability to live longer than Teff but also maintains enhanced intrinsic functionality remains despite evidence that antigen is required for protection and that effector function may be a terminal event. This hope lies in a T cell memory subset temptingly named effector memory, and is confirmed by studies of resident effector memory cells with both features [107], and protective relatively long-lived effector cells [22]. However, it is possible, as many groups have taken as dogma, that only Tcm have the capacity to live for the long-term. If so, we will have to settle for the increase in precursor specificity and the increase in proliferation capacity and speed that Tcm represent, and this approach has proven successful in some animal models [55, 83]; but not yet in attempts to design protective long-lasting T cell-inducing vaccines [42]. Therefore, by emphasizing the potential of Tem, we do not intend to detract from the value of the demonstrated fact that in the absence of excessive competition, antigen-specific memory T cells can survive in the absence of antigen, increasing the precursor T cell numbers for subsequent infections; but simply to register the possibility that perhaps Tem will surprise us in this regard and be long-lived once effectively separated from Teff. On the other hand, if short-lived Teff or Tem are required for the full protection that we seek to protect billions of children from debilitating chronic infectious diseases, then there is hope in the observation that the commonly used prime-boost strategy actually does generate cells with a Tem phenotype [251], and that boosting generates high numbers that decay less [87]. Intriguingly, high numbers of protective memory CD8 T cells have been generated in this way and shown to protect in murine malaria [83]. If they furthermore require continual stimulation for maintenance, then we currently find hope for protective T cell vaccines in the possibility of new kinds of vaccination, such as the chronic viral system developed by Hansen *et al*., as well as in the possibility that Tem may survive longer than previously thought [100, 252], a possibility that has not yet been explored enough in peripheral lymphoid organs to rule this out.
